# P-732. Clinical Utility of the Multiplex, Point-of-Care Cobas Liat CT/NG/MG Nucleic Acid Test Compared with Standard Practice

**DOI:** 10.1093/ofid/ofaf695.943

**Published:** 2026-01-11

**Authors:** Gregory Hirsch, Stephanie E Cohen, Jason Ramm, Zune Huynh, Susie Chang, Christopher Dodoo, Rodney Arcenas, Alison Cohee, Elizabeth Talmont, Louis Kuritzky

**Affiliations:** Planned Parenthood of Northern, Central, and Southern New Jersey, New Jersey, New Jersey; San Francisco Department of Public Health, San Francisco, California; Baylor Scott & White Health, Killeen, Texas; Roche Molecular Systems, Inc, Pleasanton, California; Roche Molecular Systems, Inc, Pleasanton, California; Roche Molecular Systems, Inc, Pleasanton, California; Roche Molecular Systems, Inc, Pleasanton, California; San Francisco Department of Public Health, San Francisco, California; Planned Parenthood of Northern, Central, and Southern New Jersey, New Jersey, New Jersey; Department of Community Health and Family Medicine, University of Florida, Gainesville, FL

## Abstract

**Background:**

Fast, accurate, point-of-care (POC) diagnosis can inform appropriate and timely treatment and patient management, optimizing antimicrobial stewardship and treatment outcomes compared with current standard of care (SOC) practices, including presumptive treatment. We present interim study results comparing the clinical utility of the Cobas® Liat CT/NG/MG test–a molecular POC multiplex test for detection and differentiation of *Chlamydia trachomatis* (CT), *Neisseria gonorrhoeae* (NG), and *Mycoplasma genitalium* (MG)–with current SOC.

**Figure:**
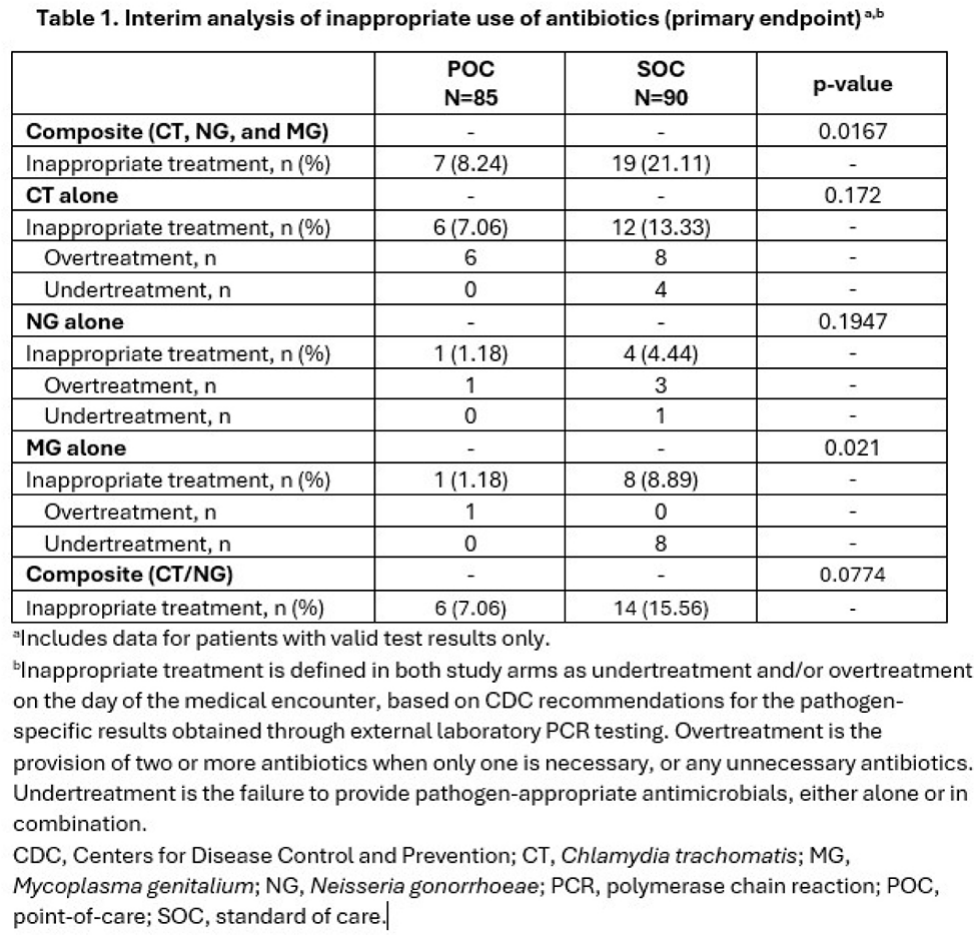

**Figure:**
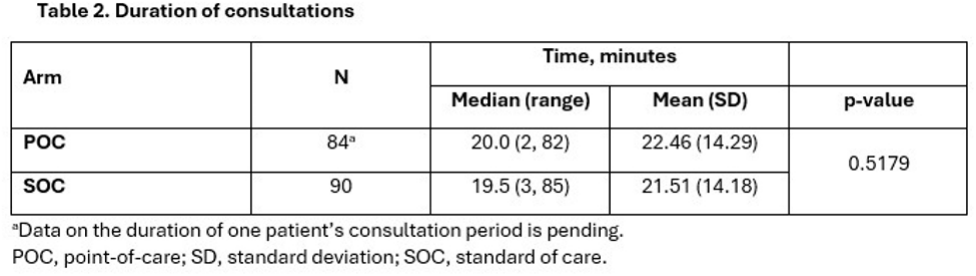

**Methods:**

This ongoing, multicenter, prospective trial is enrolling adults with exposure to or suspected sexually transmitted infection who are randomized 1:1 to SOC diagnostic testing at discretion of treating provider vs. POC test. Patients in both arms provide urogenital samples (urine or vaginal swab) for external laboratory (FDA-approved) and clinician-ordered testing. Patients in the POC arm also have a sample tested for CT/NG/MG using the Cobas Liat test and results of the POC test are provided to the treating clinician at the beginning of the visit. We compared frequency of inappropriate treatment on the same day as their medical encounter, including overtreatment (use of ≥2 antibiotics when only 1 is necessary, or any unnecessary antibiotics) and undertreatment (not providing pathogen-appropriate antimicrobials alone/in combination) between the two arms for composite endpoints (e.g., CT, NG, and MG) and for each pathogen individually. Consultation times were also compared.

**Results:**

As of December 2024, a total of 175 of the estimated 348 patients were enrolled and included in this interim analysis. The overall inappropriate treatment rate was 15% (26/175). Inappropriate treatment was less likely in the POC vs. SOC arm for composite (8% vs. 21%, p=0.0167) and MG (1% vs. 9%, p=0.021) endpoints (Table 1). Both arms had similar average consultation times (p=0.5179; Table 2).

**Conclusion:**

These interim study data show that Cobas Liat CT/NG/MG can decrease the need for presumptive treatment and reduce inappropriate treatment prescribing, providing the potential to improve patient outcomes and enhance antimicrobial stewardship.

**Disclosures:**

Stephanie E Cohen, MD, Cepheid: Grant/Research Support|Hologic: Grant/Research Support|Roche Molecular Systems, Inc: Grant/Research Support Zune Huynh, MD, Roche Molecular Systems, Inc: Employee|Roche Molecular Systems, Inc: Stocks/Bonds (Public Company) Susie Chang, PharmD, Roche Molecular Systems, Inc: Employee|Roche Molecular Systems, Inc: Stocks/Bonds (Public Company) Christopher Dodoo, MS, Roche Molecular Systems, Inc: Employee Rodney Arcenas, PhD, Roche Molecular Systems, Inc: Employee Louis Kuritzky, MD, Roche Diagnostics Solutions: Advisor/Consultant|Roche Molecular Systems: Advisor/Consultant

